# Venice as a paradigm of coastal flooding under multiple compound drivers

**DOI:** 10.1038/s41598-022-09652-5

**Published:** 2022-04-06

**Authors:** Christian Ferrarin, Piero Lionello, Mirko Orlić, Fabio Raicich, Gianfausto Salvadori

**Affiliations:** 1 CNR - National Research Council of Italy, ISMAR - Institute of Marine Sciences, Venice, Italy; 2 DiSTeBA - Department of Biological and Environmental Sciences and Technologies, University of Salento, Lecce, Italy; 3 Department of Geophysics, Faculty of Science, University of Zagreb, Zagreb, Croatia; 4 CNR - National Research Council of Italy, ISMAR - Institute of Marine Sciences, Trieste, Italy; 5 Department of Mathematics and Physics, University of Salento, Lecce, Italy

**Keywords:** Physical oceanography, Physical oceanography, Natural hazards

## Abstract

Full comprehension of the dynamics of hazardous sea levels is indispensable for assessing and managing coastal flood risk, especially under a changing climate. The 12 November 2019 devastating flood in the historical city of Venice (Italy) stimulated new investigations of the coastal flooding problem from different perspectives and timescales. Here Venice is used as a paradigm for coastal flood risk, due to the complexity of its flood dynamics facing those of many other locations worldwide. Spectral decomposition was applied to the long-term 1872–2019 sea-level time series in order to investigate the relative importance of different drivers of coastal flooding and their temporal changes. Moreover, a multivariate analysis via copulas provided statistical models indispensable for correctly understanding and reproducing the interactions between the variables at play. While storm surges are the main drivers of the most extreme events, tides and long-term forcings associated with planetary atmospheric waves and seasonal to inter-annual oscillations are predominant in determining recurrent nuisance flooding. The non-stationary analysis revealed a positive trend in the intensity of the non-tidal contribution to extreme sea levels in the last three decades, which, along with relative sea-level rise, contributed to an increase in the frequency of floods in Venice.

## Introduction

Coastal flood events are among the most disastrous natural phenomena of major risk to the safety and sustainability of coastal communities worldwide. Coastal flood risk has increased world-wide in the last decades, mostly due to mean-sea-level rise^1–4^. Coastal flooding is determined by anomalously high sea levels which are the sum of several tidal and non-tidal processes acting at different temporal and spatial scales^5^. Meso-scale atmospheric disturbances, synoptic-scale phenomena, seasonal oscillations and planetary atmospheric waves generate sea-level disturbances at different frequencies. Seiches, river floods, ocean waves, inter-annual and inter-decadal dynamics and relative sea-level rise can also contribute to the total sea level.

In this study, we analyze the long term sea-level time series recorded in the low-lying historical city of Venice (Italy), located in the northern end of the Adriatic Sea, a semi-enclosed regional basin with one of the largest tidal range (the height difference between high tide and low tide) and extreme sea levels (ESLs) in the Mediterranean Sea^6^. As a result of the devastating series of floods occurring in November 2019^7^, Venice has been defined as the “canary in a coal mine” for coastal flooding worldwide^8^, also because with 15 flood events in a month it experienced something similar to what the flooding frequency will be in the future with 30 cm of sea level rise^9^. The city of Venice represents a key study site for coastal flooding for several reasons: (i) it has a long-lasting record of sea-level observations (since 1872), (ii) Venice is frequently exposed to floods, locally called *Aqua Alta* (literally, high water), (iii) the frequency of flood events has increased over time and is likely to continue increasing in the future mainly due to sea-level rise and subsidence, (iv) it has a worldwide recognized relevance as the site is present in the UNESCO world heritage list (https://whc.unesco.org/en/list/394/), (v) an experimental and extensive flood protection plan based on the MoSE mobile barrier system has been designed (https://www.mosevenezia.eu/).

The unexpected and peculiar characteristics of November 2019 floods^7,8^ reveal the need to further explore the processes determining coastal flooding. Interestingly enough, such a phenomenon belongs to the class of so-called compound events, of utmost interest in recent geophysical research^10^. The specific objectives of the present research are to (i) investigate the relative importance of the different contributions to extreme sea levels, (ii) study their temporal change, (iii) examine their non-linear interactions and (iv) estimate the probability of occurrence of extreme events generated by the superposition of multiple drivers. In this study, Venice is taken as a paradigm of coastal flooding whereby the methodology proposed here for the analysis of extreme sea levels may be tuned and applied to other locations worldwide.

## Sea level data and physical processes at work

Regular instrumental observations of the sea level in the city of Venice began in 1871 at the tide gauge named “Punta della Salute”. The sea-level records were referenced to mean sea level (MSL) over the period 1885–1909. This datum is locally called “Zero Mareografico Punta della Salute” (ZMPS)^11^. In this study, we analyze the sea-level time series recorded in Venice from 1872 to 2019 (148 years) consisting of: the sequence of water level maxima and minima from 1872 to 1939 (generally four values per day, that is on average 6-hourly measurements); hourly values from 1940 to 2009; 10-min values from 2010 to 2019. Since October 2020, the mobile barriers at the three inlets (MoSE) have started to be in a pre-operational phase closing the lagoon during severe events and protecting Venice from flooding^12^. Therefore, this study presents the most comprehensive analyses of ESLs within Lagoon of Venice but, because of the MoSE barriers, it may not be extended into the future.

According to Orlić^13^ and Lionello et al.^14^, we here consider the sea level in Venice as the superposition of the following contributions attributable to different physical processes:

- *astronomical tide*: a mixed semidiurnal tide prevails in the northern Adriatic Sea with eight principal tidal constituents, four semi-diurnal and four diurnal^15^. The tidal range reaches almost 1 m during peak springs;
- *storm surge*: the response of the sea level to synoptic air pressure and wind forcing. In Venice, storm surge events are mostly driven by the south-easterly wind (Sirocco) blowing over the Adriatic Sea or a combination of north-easterly wind (Bora) over the northern Adriatic Sea and Sirocco over the south Adriatic Sea^16^;
- *seiches*: free sea-level oscillations in the Adriatic Sea with periods determined by the normal modes of the basin that are mostly triggered by previous storm surges when the atmospheric forcing vanishes. The decay time of these oscillations is estimated at about 3 days^17,18^. The two main modes have periods of about 21.8 and 10.7 h, which are close to the periods of the principal diurnal and semi-diurnal tidal constituents, respectively^15^;
- *meteotsunami*: large waves driven by mesoscale atmospheric pressure disturbances often associated with fast-moving weather events, such as severe thunderstorms, squalls, and other storm fronts. Such high-frequency sea-level oscillations are generated by resonance in the open sea when the speed of propagation of the perturbation approaches that of the shallow-water barotropic waves. In harbours and bays, coastal resonance may also play an important role in the local amplification of meteotsunami waves^19^;
- *local wind setup within the lagoon*: with strong NE (Bora) or SE (Sirocco) winds, the difference between sea levels in the south and the north side of the lagoon may exceed 50 cm^20^. Even if Venice city centre is little affected by these fluctuations, since it is close to the node of the oscillation of the water level in the lagoon, a high-fequency wind setup (of the order of 10 cm) has been observed under strong SW winds^7^;
- *PAW surge*: long-lasting sea-level anomalies generated by disturbances of air pressure and wind associated with planetary atmospheric waves (PAW) having characteristic wavelengths ranging from 6000 to 8000 km^21^. These events may result in a prolonged interval of high sea level in the northern Adriatic Sea, which provides the long-term preconditioning for many flood events in Venice^22^;
- *inter-decadal, inter-annual and seasonal (IDAS) sea-level variability*: the occurrence of ESL also displays marked seasonal to decadal variability associated with large scale atmospheric and oceanic circulation patterns^9,15^. According to Valle-Levinson et al.^23^, interannual variability of MSL in the northern Adriatic can be mostly explained by astronomic forcing associated with lunar precessions, solar activity, and the interaction, or interference, between these factors;
- *relative sea-level rise (RSLR)*: a long-term process connected to both climatic change and land subsidence^9^. In the 1872–2000 period, the combined effect of the subsidence and sea-level rise resulted in a relative rise of 31 cm^24^, determining a substantial increase in the frequency of floods in Venice^16^. Mean sea level of 2019 is 0.34 m above ZMPS.

All listed contributions have values of the order of tens of centimetres (storm surge is potentially the largest one, exceeding one meter in extreme cases). Other factors, like river floods, earthquake tsunamis and waves are not relevant for the sea-level variability in Venice^14^. For simplicity, the combination of the meteorological contributions with periods lower than 10 days excluding the seiches (storm surge, mesoscale atmospheric variability set-up including meteotsunamis and local wind setup) is collectively termed “storm surge” in this paper when no distinction is possible among the different drivers.

## Results and discussion

### Identification of extremes

In this work, we are interested in extreme sea-level events driven by any combination of the tidal and non-tidal components. A peak over threshold method is often used to identify extreme sea levels from tide gauge observations^25^. However, selecting an appropriate threshold is still challenging and can bias the statistical assessment. In Venice, a value of 1.40 m above ZMPS is the threshold commonly used for identifying exceptional events (https://www.comune.venezia.it/it/content/le-acque-alte-eccezionali). However, with such a high threshold few data sets (25 events in the 1872–2019 period^26^) would be included in the analyses. Other arbitrary thresholds can be used, such as a water height of 1.10 m above ZMPS, which is the height at which the MoSE barriers will be activated to prevent the flooding of the historical city^12^.

**Figure 1:**
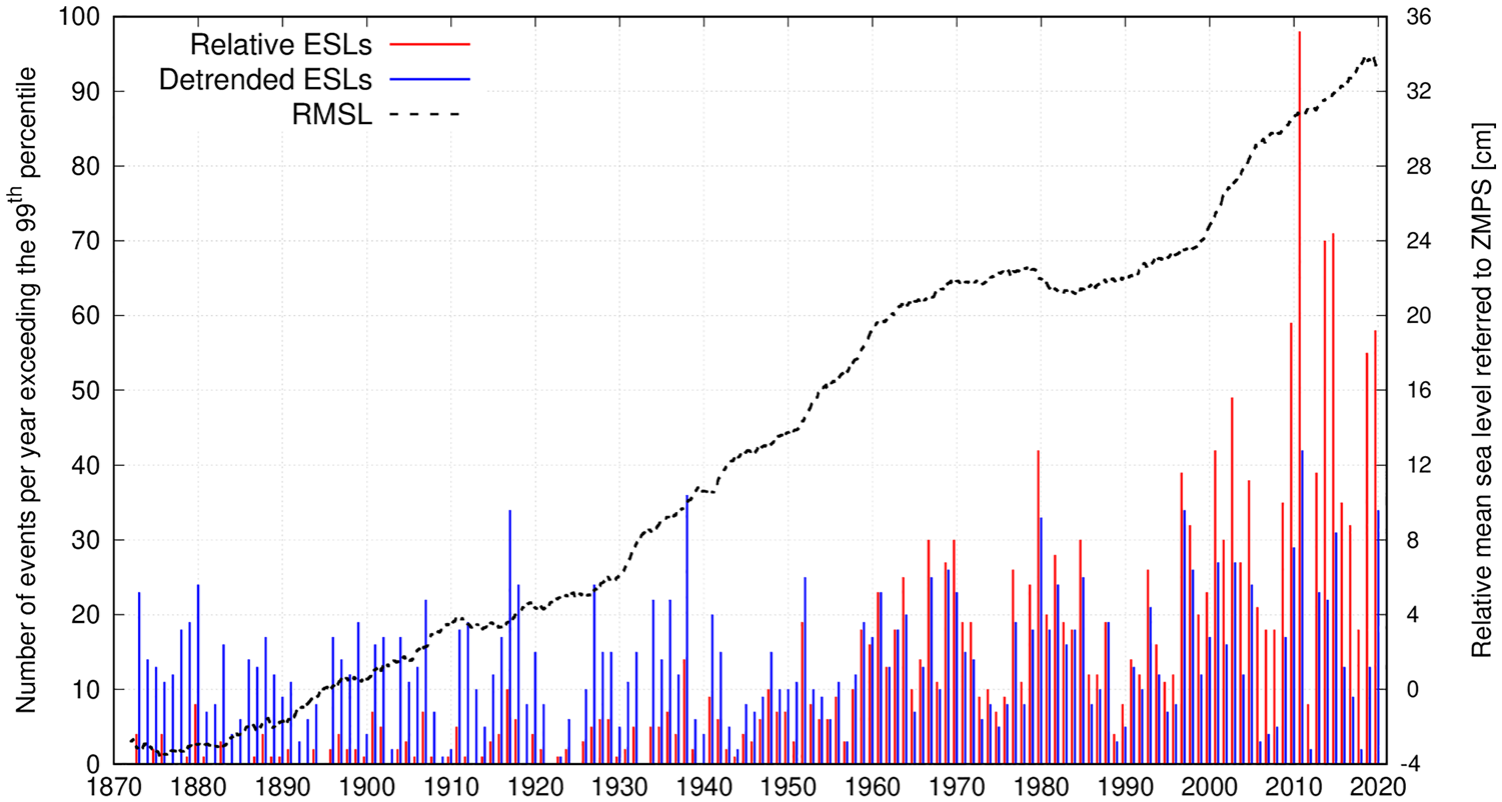
Number of events per year exceeding the 99th percentile threshold in the relative (red bars) and detrended (blue bars) sea-level datasets. The time evolution of the relative mean sea level (19-year running mean) is shown as a black dashed line.

Relative sea level in Venice has been strongly influenced (i.e. raised) by subsidence and eustasy (1.30 and 1.23 mm/year on average over the 1872–2019 period, respectively^9^). Therefore, the extreme value selection was based on the distribution of the sea levels detrended for RSLR by subtracting the 19-year centered running mean (black dashed line in Fig. 1). Then, the 99th (0.70 m), 99.5th (0.77 m) and 99.9th (0.95 m) percentiles of sea levels are used here for identifying extreme values (2012, 1005 and 201 events, respectively). As shown in Fig. 1, the detrended dataset presents a much more homogeneous distribution of ESL events over time with respect to the relative (uncorrected for RSLR) sea-level dataset.

### Drivers of extreme sea levels

Tidal harmonic analysis and digital filters were used to separate the sea-level time series into different water level components (tide, storm surge, high-frequency surge, seiches, PAW surge, IDAS). A detailed description of the decomposition procedure is reported in the Methods section. Extreme sea levels in Venice turned out to be driven mostly by the non-tidal residual (i.e. $>50$%) with the astronomical tide contributing on average 49, 44 and 36% of ESLs when considering the 99th, 99.5th and 99.9th percentile datasets, respectively (Table 1). These results agree with other studies^26^ showing that the highest sea levels are mainly dominated by the non-tidal residual (NTR), while tide plays an important secondary role.

**Table 1:** Mean contribution (in %) of the different drivers of ESLs defined according to the 99th, 99.5th and 99.9th percentile datasets. Tide and NTR contributions are computed with respect to the total sea level, while the other non-tidal contributions are computed with respect to NTR.

Driver of ESL		99th	99.5th	99.9th
Astronomical tide		49	44	36
Non-tidal residual (NTR)		51	56	64
NTR	Seiche	14	15	16
	Storm Surge	29	34	44
	PAW surge	40	37	29
	IDAS	17	14	11

**Figure 2:**
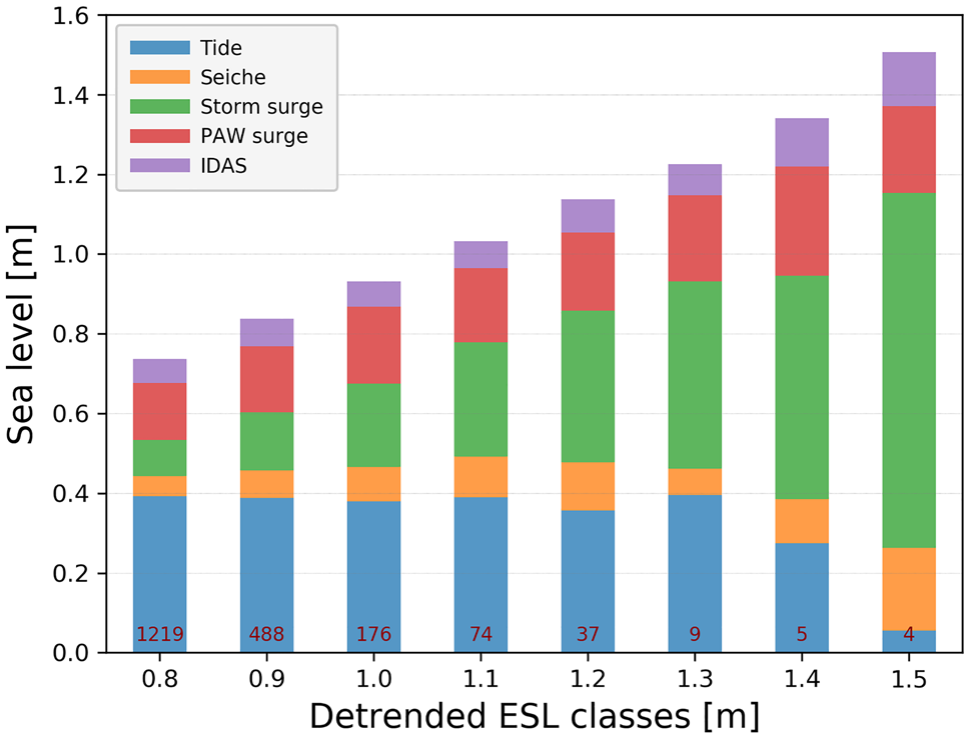
Mean contribution (in m) of the different drivers of ESLs subdivided into 10 cm bins. The red labels inside the bars indicate the number of events per class.

The non-tidal residual of the extreme sea levels could be attributed mostly to PAW surge and storm surge, with the latter component becoming dominant for the most extreme events. In general, the relative role of low-frequency signals (PAW surge and IDAS) decreases with the severity of the event, and the opposite occurs for the short term components (with periods shorter than 10 days: seiche and storm surge). However, as illustrated in Fig. 2 which reports the relative contribution of the drivers for different values of the ESLs, this does not correspond to a decrease of the absolute values of the PAW surge and IDAS terms, the sum of which, on average, increases from 20 to almost 40 cm towards the most severe events. Positive IDAS ($\max = 0.26$ m) and PAW surge ($\max = 0.43$ m) oscillations could result in a prolonged interval of high sea level in the northern Adriatic Sea, as observed in November 2019^7^. These results confirm previous studies highlighting the important role of low-frequency fluctuations in providing favourable conditions for flooding events in the northern Adriatic Sea^22^. Such a strong contribution of the low-frequency oscillations to the Venice floods emerged in the last decades in association with the high mean relative sea level. For more details about the inter-decadal, inter-annual and seasonal sea-level variability, the reader may refer to the review paper by Zanchettin et al.^9^.

The storm surge contribution varies from 0.10 to 0.70 m with a clear positive trend towards the most extreme events. The highest storm surge event (1.07 m) occurred on 4 November 1966, when the total sea level reached the maximum value ever recorded in Venice (1.94 m above ZMPS). Other events with extreme storm surge contributions occurred in 1979 (0.75 m), 2018 (0.74 m) and 1879 (0.74 m). All these events were driven by strong SE winds blowing over the whole Adriatic Sea and determining the rise of the sea level and high waves in the northern end of the basin^16^.

On average, seiches have a contribution that increases in magnitude from 0.05 to 0.21 m with the severity of the event (Table 1, Fig. 2). Seiches are responsible for extreme sea-levels usually after a substantial surge event, which triggers fundamental oscillations of the Adriatic basin. Indeed, high seiches were registered in the days succeeding the 4 November 1966 and other storm surge events^17^. However, a very high seiche oscillation (0.43 m) occurred in December 2019 without a substantial storm surge. It was probably triggered by a SE wind followed by an NW wind after about 10–11 h (i.e., half of the period of the fundamental Adriatic seiche). Such resonant excitation with two relatively week impulses, but perfectly timed (i.e. a typical compound event), caused an oscillation which represented more than half of the non-tidal residual signal.

The 1940–2019 hourly time series only was used for analyzing sea level high-frequency fluctuations with a period lower than 10 h. The maximum value of this component (0.36 m) was registered on 12 November 2019, due to the combined action of a meteotsunami propagating along the coast (0.27 m) and a local surge inside the lagoon (0.09 m), both induced by a meso-beta scale atmospheric disturbance travelling along the north-eastern Adriatic coast^7^. The database shows no evidence of other high-frequency events of similar magnitude and only six events with high-frequency surges equal to or higher than 0.15 m were identified. Therefore, according to the analysis of the 1940–2019 dataset, the meteotsunami that hit the coast in front of Venice on 12 November 2019 represents an extremely rare episode.

#### Non-stationary analysis

**Figure 3:**
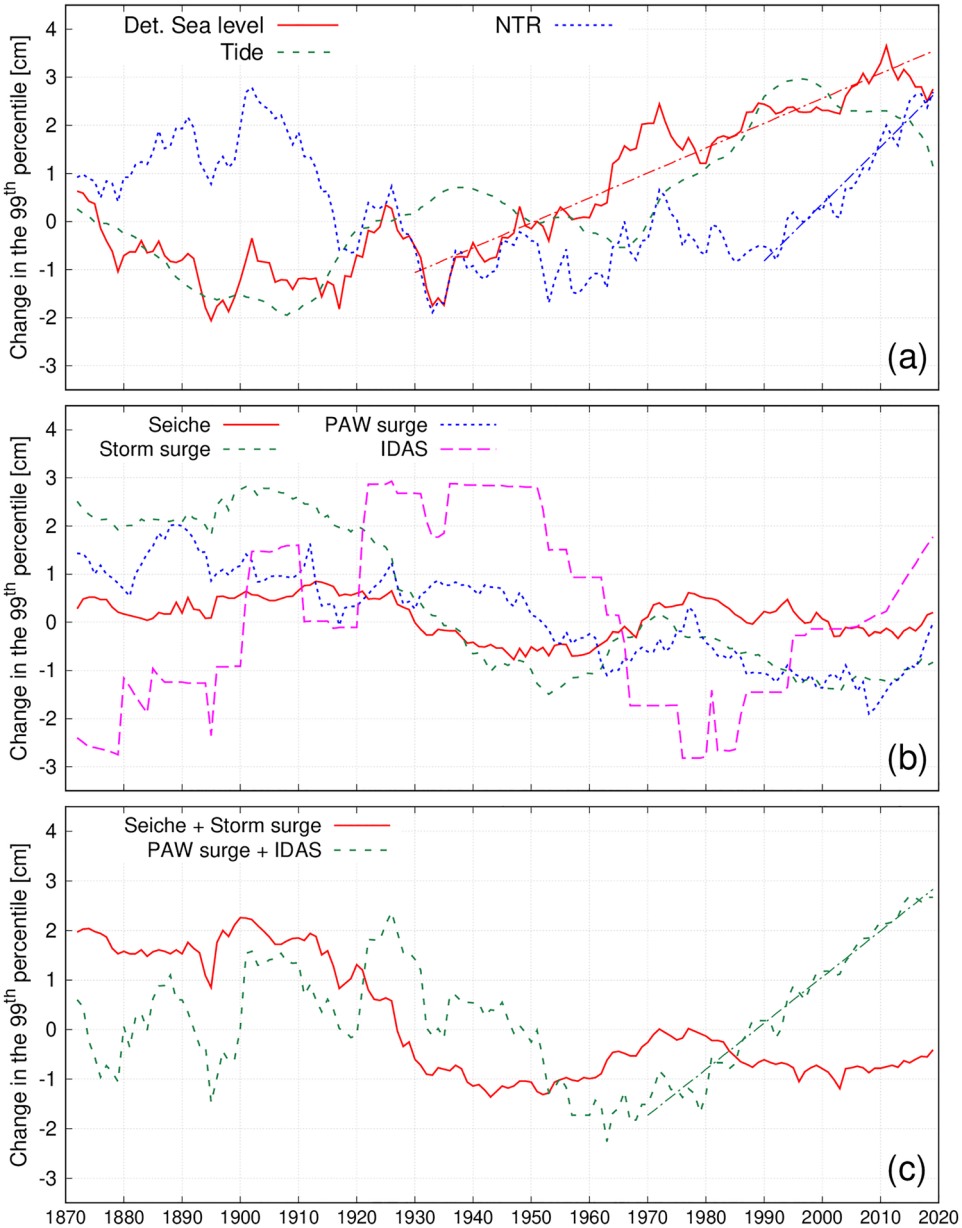
Temporal changes of the 99th percentiles of the different drivers, computed with respect to the long-term mean value. The dash-dotted lines indicate the trends discussed in the text (slope statistically significant at the 0.05 level).

Temporal changes in extreme sea levels over the 1872–2019 period were investigated by computing the 99th percentile of sea level maxima on a 30-year centered running window^25^ (Fig. 3a). Orlić et al.^27^ showed that the 30-year window suppresses the decadal-scale variability of the Adriatic sea levels while it does not drastically reduce the length of the time series. This implies that the multidecadal variability remains in the filtered time series and has to be allowed for while interpreting the results obtained from them. The 99th percentile of the detrended sea levels (red line) fluctuated around a constant value from 1872 to 1930 and then subsequently increased. We also investigated the 99th percentile evolution of the different contributions, which, even if their values cannot be summed linearly, may provide information on the temporal changes of the processes at work. Changes in the 99th percentile of the tidal (green dashed line) and non-tidal residual (blue dotted line) components showed almost opposite behaviours with a decrease (increase) phase from 1872 to 1910, an increase (decrease) phase till 1990 and then a decrease (increase) phase again in the last decades. The general increase in the amplitude of major tidal constituents is mostly due to the anthropogenic interventions that altered the lagoon’s morphology during the last two centuries (salt marsh reduction, dredging of navigational channels, groundwater and gas extraction, inlets’ modifications). These morphological changes resulted in a reduction of the dissipative forces due to friction, with the consequent amplification, due to resonant processes, of the incoming tidal wave while it propagates inland^28^. Changes in the detrended NTR 99th percentile—which includes the contribution of storminess (storm surge) and flood preconditioning (PAW surge and IDAS)—show an increasing trend in the last three decades.

Slightly negative tendencies of the 99th percentile can be identified for the storm surges and the PAW surges, while no clear long-term trend can be detected for the other components (Fig. 3b). Interestingly enough, the analysis of the percentile evolution of the combined contributions referring to short-term (period $< 10$ days, i.e. seiche plus storm surge) and long-term (period $> 10$ days, i.e. PAW surge plus IDAS) revealed that the increase of the 99th percentile of the non-tidal residual in the last three decades seems to be mostly due to changes in the IDAS and PAW surge (Fig. 3c), the combination of which determines the large-scale dynamical precursor for flooding in Venice. The PAWs, and the related PAW surges, could change considerably with the global warming: since the latter is much more pronounced in the polar than in the equatorial regions, the amplitudes of PAWs increase and their speeds decrease^29^ implying that the amplitudes of PAW surges increase and that these surges last longer. The seasonal analysis of the 99th percentile revealed that the data of October, November and December (when most of the flooding events occur^16^) have a more marked increasing trend for both the detrended sea level (8 cm since 1880) and the NTR component (about 10 cm since 1930). The detected changes in the extreme values of the different components are relatively small (within a few cm for all terms) when compared to changes in relative mean sea level (about 0.37 m in 148 years; black line in Fig. 1).

#### Non-linear interactions (NLI)

Even if surges can occur in association with any tide, tidal and non-tidal components have a certain degree of interaction in shallow water areas with large tidal excursions where shoaling and other non-linear effects are considerable^30,31^. Therefore, tides and storm surges may mutually affect each other’s phase and this interaction may lead to high surges often occurring during low water and before high water (HW) than at HWs^30,32^ during flood tides. Moreover, wind stress is more effective at raising the sea surface in shallow water and therefore one would expect any surge to be largest at low water^30^.

**Figure 4:**
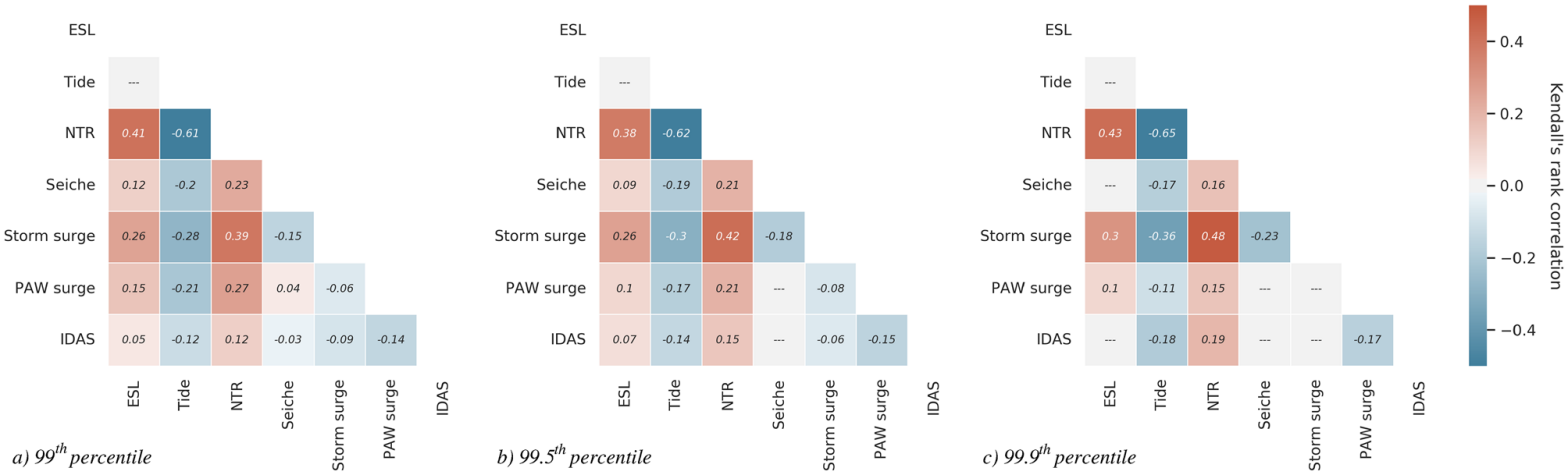
Kendall’s rank correlations among the different drivers for the 99th (**a**), 99.5th (**b**) and 99.9th (**c**) ESL percentiles. Only values statistically significant at the 0.05 level are reported.

The statistical correlation between tide and non-tidal residuals obtained decomposing tide gauge records has often been used as a measure of the influence of non-linear interactions on extreme sea levels^25,33^. Our statistical analysis, even if performed over a longer timeseries than used in previous studies, produced similar results showing a marked negative correlation between tide and NTR which slightly increases with the severity of the events. Indeed, the non-parametric Kendall’s rank correlation $\tau$, an association measure ranging between $(-1,1)$, takes on values of $-0.61, -0.62$ and $-0.65$ when considering, respectively, the 99th, 99.5th and 99.9th percentiles as thresholds for identifying extreme sea levels (Fig.4): this means that the variables are statistically *discordant*, i.e. small (large) values of one variable are likely to be associated with large (small) values of the other, and vice-versa. Tides were found to be negatively correlated with all factors affecting extreme sea levels. Seiches are anticorrelated with storm surges, which trigger free oscillations in the Adriatic Sea. However, since the decay time of these oscillations is 3–4 days, they can overlap with a new storm surge causing very high sea levels in the northern Adriatic Sea (e.g. on 1 December 2008 and 25 December 2009^18^). Therefore, even if storm surges and seiches are generally out of phase (anticorrelated), joint occurrences of these two factors are possible since the passage of synoptic systems can take place in rapid succession. The different meteorologically-induced sea-level signals (storm surge, PAW surge, IDAS) were weakly ($|\tau | < 0.2$) or not correlated with each other, highlighting that, from a statistical perspective, the atmospheric processes evolving at different spatial and temporal scales (mesoscale, synoptic, planetary) act almost independently on the extreme sea levels in Venice.

In this study, we used the Theory of Copulas^34,35^ to identify an appropriate model to capture the dependence structure between the mentioned drivers and combine their joint occurrences to calculate extreme sea levels. We considered ESLs as determined by the sum of tide and NTR, assuming the two variables either (i) as dependent and modelled via a suitable bivariate copula, or (ii) as being independent. Similarly, a multivariate analysis has also been performed considering the ESL determined by the combination of tides, seiches, storm surges, PAW surges and IDAS. Sea level percentiles (99th, 99.5th and 99.9th) are calculated for the dependent and independent artificial samples (for both the 2 and 5 variables cases) and differences between these values are assumed to describe NLI on ESLs^25^. The estimated tide-NTR NLI is sensitive to the considered dataset and ranges from $-26$ to $-52$ cm. Similarly to the case with two variables, the NLI estimated from the Copula functions with five variables ranges from $-23$ to $-45$ cm. The largest values were found for the most severe events (Supplementary Table S1).

These anti-correlations apparently suggest that some non-linearity is at work in the northern Adriatic Sea and the Lagoon of Venice with the tide having a lowering effect on extreme sea levels. In the northern Adriatic Sea, given the relatively small importance of tidal excursion (about 1 m) as compared to local water depth (average depth of about 35 m), it is reasonable to neglect the effect of tides on storm surge generation and propagation. Similarly, storm surges would not significantly influence the tide. These assumptions have been confirmed by high-resolution numerical studies demonstrating that tide-NTR interactions are negligible in the northern Adriatic Sea, even during the most severe events^14,36,37^. Moreover, a similar tide-NTR anticorrelation was found by analyzing the 1974–2019 sea-level dataset collected by the Acqua Alta oceanographic tower, located in the Gulf of Venice, 15 km offshore the Venetian littoral. Sea-level oscillations in the storm surge frequency band, once they have entered inside the lagoon through the inlets, propagate nearly without modification to the Venice city centre, where residual levels are comparable (apart from the local effect of the wind) to the ones at the inlets with a typical 1-hour delay^38^.

The tide-NTR anticorrelation found by the statistical analysis could not, therefore, be fully explained by ocean non-linear processes and results from the fact that, generally, the highest NTRs tend to occur around mid-tide or low-tide rather than at the time of tidal high water. Indeed, in the most extreme storm surge events of 1966, 1979 and 2018, the peak of the storm occurred during low tidal conditions, limiting the already dramatic flooding conditions in Venice. Even if the peak astronomical tides and peak storm surges tend to occur in different parts of the year in the northern Adriatic Sea^32^, the observed tide-NTR anticorrelation is an unresolved issue and a clear ocean process-based explanation of it is still missing.

### Probability of occurrence of ESLs

**Figure 5:**
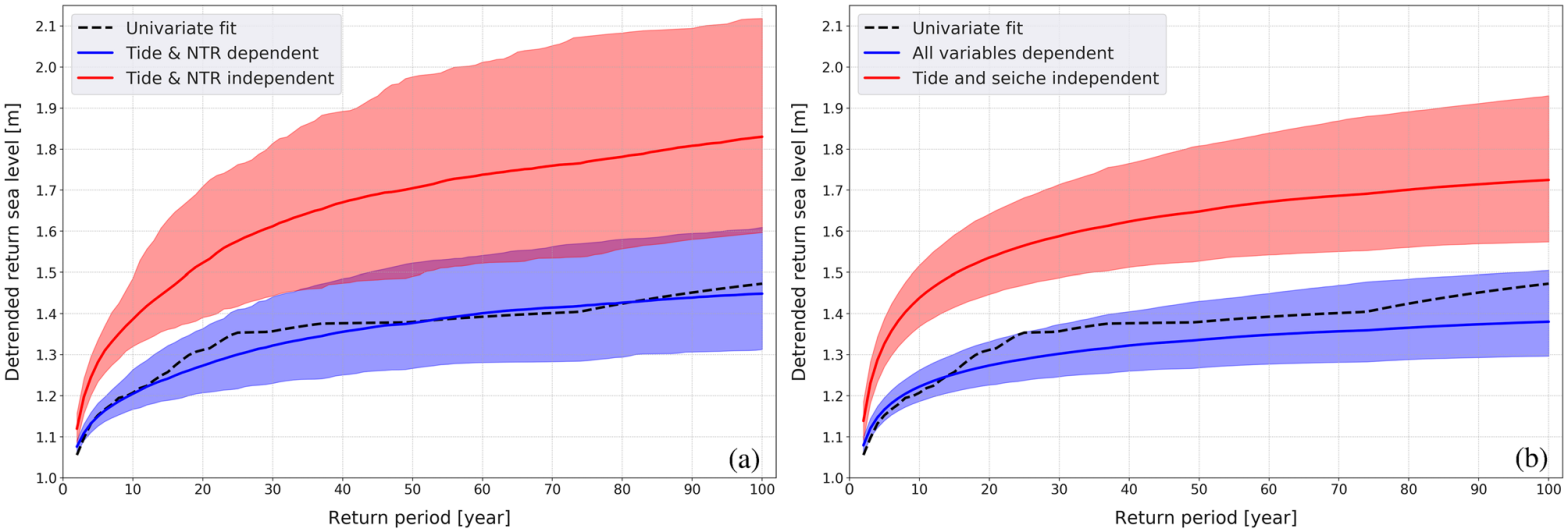
Return sea levels and periods obtained from the univariate fit of ESLs and as modelled considering the combination of two (panel **a**) and five (panel **b**) variables. Model’s median (solid line) and 95% confidence interval (band) are shown.

The results presented in the previous sections were used to investigate the probability of occurrence of an extreme sea level generated by the combination of the different processes. For return periods (RP) varying from 2 to 100 years, the corresponding design values of ESLs are calculated by aggregating the variables of the simulated vectors of marginal components under the dependent and independent assumptions (Fig. 5). We only present the results of the models based on the 99.9th percentile dataset, which best fits the observations, supporting the fact that the degree of association among the drivers extracted from the most severe events are most appropriate for representing the dynamics of ESLs in Venice Lagoon.

The return sea level determined from the tide-NTR dependent model (blue continuous line in Fig. 5a) well represents the RP statistics extracted from the univariate fit of the detrended sea levels (black dashed line). If the non-linear tide-NTR interactions are not considered (red continuous line in Fig. 5a), the return sea levels are overestimated, which means that extreme sea levels could occur with a higher frequency. As an example, a sea level of 1.4 m would have an RP of about 10 years in the independent case and an RP of more than 100 years in the dependent case. Similar results were obtained considering the aggregation of five variables (tide, seiche, storm surge, PAW surge, IDAS) (Fig. 5b). It is worth noting that, on the one hand, the 5-variables model may account for the simultaneous (compound) effects of the five drivers of interest, and, on the other hand, it has narrower confidence bands with respect to the 2-dimensional (tide-NTR) model.

These results highlight the large influence of the non-linear interactions—mostly linked with the effect of the tide with the other components—on the return sea levels and related occurrence periods. Moreover, there is a large variability in the RP’s estimation using different datasets, as highlighted by the values computed for some of Venice’s most extreme sea-level events (Supplementary Table S2). To compute the RP of such extreme values, the ESL data have been fitted with different models (see the Methods section) choosing the one that best represents the data. The 1966 event has a return period ranging from 255 to 929 years according to the dataset used in the calculation. The 356 years RP of the 1966 events estimated using the 99.9th percentile dataset is consistent with previous estimations^32,39^. The flooding event of November 2019—second only to the 1966 event—should occur on average once every 92 years.

### ESLs and coastal flooding

Every coastal location has site-specific flooding thresholds that can change due to oceanographic drivers, as well as changes in flood defences and coastal morphology^40^. In the ESL analysis presented above, we considered the detrended sea levels for investigating the dynamics of extreme events removing long-term subsidence and eustatic effects. However, when dealing with coastal flooding, the ESL values must be referred to the local relative sea level. The probability of occurrence of a specific flooding level can be easily calculated by shifting the return level/period curves (Fig. 5) to consider past and near-future changes in the relative sea level^3,4,41^. Therefore, the return period of the 1966 flood, which reached the peak water height value of 1.94 m above ZMPS (about 90% of the city flooded), reduces to less than 100 years when considering a relative sea-level 30 cm higher than today. Considering less extreme events, the return period of a water height value of 1.40 m above ZMPS (60% of the city flooded) changed from about 70 years at the beginning of the 19th century to less than 5 years today.

It is important to highlight that the relative role of the different drivers in coastal flooding changes according to the mean relative sea level and the flooding threshold considered. We used to focus our attention on the most extreme events and their consequences on coastal flooding. As shown above, these events were mostly driven by storm surges (e.g. 1966, 1979 and 2018 events). However, it is noteworthy that in Venice even a low level of flood (i.e. nuisance flooding^42^, determined by water heights between 1.10 and 1.40 m above ZMPS) has high impacts such as disruption of everyday routine activities and property damages. Such events can be caused by several drivers or their combination (e.g. the sequence of exceptional floods of November 2019^7^), thus increasing the probability of flood occurrence. Indeed, use of different thirty-year time windows revealed:

- 8 nuisance floods in 1890–1919, half dominated by tide (i.e. > 50% of the total sea level) and half by storm surges;
- 44 nuisance floods in 1940–1969, one fifth of which were dominated by storm surges, and tides and long-term fluctuations (IDAS plus PAW surge) representing the largest factor in 73% and 7% of the cases, respectively;
- 172 nuisance floods in 1990–2019, 70% of which were mainly driven by tide, while the long-term fluctuations and storm surge are dominating in 18% and 12% of the cases, respectively.

It is clear that in Venice, as well as in other parts of the world, SLR and subsidence lead to an increase in recurrent flooding at high tide^14,43^. Moreover, secular amplification of tides induced by RSLR and anthropogenic activities also contributed to increasing the flood frequency^28,44^. Therefore, flood risk in Venice increased during the last century and will certainly worsen in the future due to RSLR and especially if the positive trend in the large-scale precursor for flooding (IDAS plus PAW surge; Fig. 4) persists. Even if most of the climatological studies predict attenuation of storm surges^14,45^, the number and the duration of flooding events in Venice will increase in the future (exponentially according to Taherkhani et al.^4^), with high impacts on the management of flood defence measures. With a SLR of 50 cm, the MoSE barriers will have to be activated almost every day^4,12^, because the tide alone will result in a sea level exceeding the 1.10 m flooding threshold. It is worth stressing that the novel multivariate statistical framework outlined in this work may also provide useful information for improving the short term forecasts for Venice, which are especially important for the operation of the MoSE barriers^46,47^.

## Conclusions

The major conclusions of this work are summarized in the following points.

1. The spectral decomposition of sea level allows for the quantification of the relative role of the different drivers to ESLs. In the northern Adriatic Sea, storm surges are responsible for the most extreme sea levels but the drivers of coastal flooding in Venice are many and may occur together as compound events. Due to the rise of relative mean sea level, the tide and long-term meteorological components actually play a dominant role in driving recurrent—even unexceptional—floods.
2. The non-stationary analysis revealed an increasing trend in the intensity of the non-tidal contribution to the sea level in the last three decades. Such an evolution could be mostly attributed to changes in IDAS and PAW surges, the combination of which determines the large-scale dynamical precursor for flooding in Venice.
3. Copulas, which account for the statistical dependence among the different contributions to sea level extremes in case of compound events, provide an effective tool for their description, which improves the information given by univariate extreme value analysis. The analysis revealed evidence of significant correlations among the drivers, which have a lowering effect on extreme sea levels and are fundamental for producing a correct estimate of extremes.
4. Statistical correlations derived from tide-gauge records may be misleading in the evaluation of non-linear interactions in shallow water and should not be used to estimate NLI influence on extreme sea levels. In Venice, the tide-NTR anticorrelation results from the fact that, generally, the highest NTRs tend to occurs around mid-tide or low-tide rather than at the time of tidal high water. This topic will need to be further investigated and its understanding is essential from a climate change perspective in which the drivers of ESLs may have a different rate of change.

There is a massive global scientific effort in understanding and managing coastal flooding, one of the main threats to coastal communities especially in the most vulnerable low-lying areas of the world. Many coastal locations, such as Venice, are already experiencing an alarming growth of flood threat due to global warming and consequent rise of the level of the oceans. It is likely that within the next decades what we now consider to be extreme events will happen at every high tide. Obviously, the flood risk strongly depends on site-specific coastal characteristics and protection measures, but also on the processes determining the intensity of the hazards. In this context, Venice is a very important paradigm for coastal flood risk, also because it is already affected by both high-frequent nuisance and rare extreme flood events. The general methodology adopted in this study (not specific for the Lagoon of Venice only) provides new insights into the physical drivers of the extreme sea levels and therefore could add fundamental knowledge to science-based and sustainable flood management strategies all around the world.

## Methods

### Sea level decomposition

The whole dataset was first interpolated with a monotonic cubic method at 10 min interval and then detrended with a 19-year centered running mean to remove the long-term variability induced by sea-level rise, subsidence, multidecadal variability and non-linear tidal effects of the lunar cycle within the lagoon^28^. As reported by Pirazzoli et al.^32^, in this manner extreme sea levels from different years are made “climatically homogeneous” and can easily be compared in spite of relative sea-level changes^9^.

The detrended dataset was then processed with a tidal harmonic analysis tool based on the least-squares fitting^48^ to separate the tidal from the total sea level. The method considers standard 67 tidal constituents and can deal with an incomplete series of data and with observations at different time intervals. In this study, the whole 1872–2019 dataset has been analysed considering a 19-year running window to account for changes in the tidal characteristics. As reported in Ferrarin et al.^28^, the amplitude and phase of the principal tidal components in Venice exhibit a substantial change over the last century, mostly due to anthropogenic interventions. Nodal correction is considered in the processing. At each time step, the difference between the observed total sea level and the tidal value is here called non-tidal residual (NTR).

Once the decomposition of the total sea level has been carried out, we aim to identify the different non-tidal processes based on their characteristic periods^7,14,49^. The contributions of storm surge, PAW surge, mesoscale atmospheric variability (MAV) including meteotsunami, local surge, seiche and IDAS variability were isolated from the detrended NTR using digital filters (low pass, band-pass and high pass) in the time domain exploiting Fourier decomposition. The value separating storm surge and PAW surge was set to 10 days and the corresponding value for storm surge and high-frequency surge (associated with MAV set-up, meteotsunami and local surge within the lagoon) was set to 10 h. The separation between the PAW surges and IDAS variability was achieved by applying a low pass filter with the cut-off period equal to 120 days. The storm surge signal (having a period between 10 hours and 10 days) was further band-pass filtered to extract the seiche activity. These filters are symmetric around the periods of the first and second modes of the Adriatic seiches, set to 21.8 and 10.7 h, respectively^17^. The reader is referred to Lionello et al.^14^ and Ferrarin et al.^7^ for a more detailed description of the aforementioned sea level decomposition analysis.

According to the sampling theorem, the minimum period resolved by the data is twice the sampling interval. Moreover, oscillations of smaller periods may be aliased to larger periods (e.g. an oscillation having a period of 5 h would appear as a false signal at a period of 30 h in 6-hourly time series). In other words, by using a sampling interval of 6 h we would be unable to document oscillations of periods smaller than 12 h and, furthermore, if such oscillations do exist in the original time series they would contaminate the signals having periods larger than 12 h. Therefore, due to the low sampling interval of the 1872–1939 dataset, the contribution of the high-frequency surge to the sea level (period < 10 h: MAV set-up, meteotsunami and high-frequency local surge) was computed only for the 1940–2019 timeseries.

In order to ensure serial independence among individual extreme events, the original time series was subsampled to obtain a homogeneous high and low waters time series for the whole 1872–2019 period (203,100 values, approximately two high and two low waters per day). The subset correctly represents the time evolution of the long-term relative sea-level rise, as well as the tidal and seiche extremes. Indeed, the Kolmogorov-Smirnov, Andreson-Darling and Mann-Whitney tests confirmed that for the storm surge, PAW surge and IDAS contributions, the distribution of the high-low subsample does not differ (with statistical significance, p-value $> 0.05$) from the one of the original datasets. The tests were not successful for the high-frequency oscillations, but the subsample contains the most significant events (considered as the ones with values higher than 0.15 m).

### Extreme value analysis

The following analysis is based on the notion of Hazard Scenario as the upper set of the events above a given threshold^50^, e.g. the ESL larger than some critical limit (see also the Supplementary Information). The probability $\alpha$ of events belonging to a Hazard Scenario is typically small, so that they are characterized by return periods $RP = \mu /\alpha$ generally longer than $\mu$, where $\mu$ is the average inter-arrival time between successive occurrences in the time series. Such an approach can be applied both for univariate and multivariate analyses. In our analysis, the Hazard Scenario is the set of events above a sea level threshold representing a high percentile of the overall distribution.

The multivariate analyses carried out in the present work are based on the mathematical Theory of Copulas^34,35^. A copula is a multi-dimensional function that models the statistical dependence (or independence) among a set of variables of interest, which in our case are the different contributions to the sea level anomalies. Copulas are used to provide the overall probability of a compound critical event accounting for both the marginal random behavior of the variables involved and their joint statistical interactions (e.g., when considering the ESL as the aggregation of several non-independent drivers).

The very first step in any multivariate analysis is to check the *degree of association* (viz., concordance or discordance) between pairs of variables (e.g. via the non-parametric Kendall’s rank correlation $\tau$). Should two variables be regarded as dependent, then a number of parametric copulas^34,35^ could be used to model their dependence structures. Thanks to Sklar Representation Theorem^51^, in order to construct a full statistical model of the random behaviour of the variables of interest, it is necessary and sufficient to individuate the marginal distribution functions of the single variables at play, as well as their copula(s). Several standard univariate distributions and different families of copulas have been used for the univariate and multivariate fits.

In this study, first a bivariate copula $C_2$ is fitted over the available pairs of (tide, NTR). Then, $M=10,000$ artificial independent samples of the vector (tide, NTR) are randomly generated from the fitted model. The analysis is then repeated but assuming that tide and NTR are completely independent, i.e., NTR maxima can occur at any phase of the tide. A set of $M=10,000$ artificial extreme sea levels is constructed aggregating tide and NTR (i.e., excluding the NLI). Furthermore, a 5-dimensional dependence model $C_5$ is fitted assuming that tide, seiche, storm surge, PAW surge and IDAS are all dependent. However, as for tide, seiche can also be considered as an independent factor contributing to the sea level. Therefore, we could consider the pair (tide, seiche) as independent of the triple (storm surge, PAW surge, IDAS). In turn, a 3-dimensional dependence model $C_3$ is fitted over the triple, and a full 5-dimensional copula $C^{'}_{5}$ is constructed as^35^ $C^{'}_{5} (u_1, u_2, u_3, u_4, u_5) = u_1 \cdot u_2 \cdot C_3 (u_3, u_4, u_5)$, where $u_i \in [0, 1]$, with $i = 1,\ldots ,5$. This guarantees that the pair and the triple of variables considered are statistically independent. As above, $M=10,000$ artificial independent samples of the vector are randomly generated from the fitted models.

A more detailed description of the approach for the precise definitions of Hazard Scenario and Return Period is reported in the Supplementary Material.

## Supplementary Information

Supplementary Information.

## Data Availability

The sea-level data used in this study can be requested to the Centro Previsione e Segnalazione Maree - Protezione Civile, Venice, Italy, https://www.comune.venezia.it/it/content/centro-previsioni-e-segnalazioni-maree.
