# Supplementary material for: Venice as a paradigm of coastal flooding under multiple compound drivers
Source: Sci Rep. 2022 Apr 6;12:5754. doi: 10.1038/s41598-022-09652-5 (PMC8986792; doi:10.1038/s41598-022-09652-5)
Supplement: Supplementary file 1 — Supplementary Information. [file 41598_2022_9652_MOESM1_ESM.pdf]

Venice as a paradigm of coastal flooding under multiple compound drivers

Christian Ferrarin[1,*], Piero Lionello[2], Mirko Orlić[3], Fabio Raicich[4], Gianfausto Salvadori[5]

[1] CNR - National Research Council of Italy, ISMAR - Institute of Marine Sciences, Venice, Italy

[2] University of Salento, DiSTeBA - Department of Biological and Environmental Sciences and Technologies, Lecce, Italy

[3] Department of Geophysics, Faculty of Science, University of Zagreb, Zagreb, Croatia

[4] CNR - National Research Council of Italy, ISMAR - Institute of Marine Sciences, Trieste, Italy

[5] University of Salento, Department of Mathematics and Physics, Lecce, Italy

* corresponding author: Christian Ferrarin (c.ferrarin@ismar.cnr.it)

SUPPLEMENTARY MATERIAL

Supplementary Table S1. Extreme sea levels (in m) as estimated from the copula models (under the dependent and independent assumptions) using the 99th, 99.5th, 99.9th ESL datasets and corresponding non-linear interactions (in m).

ESL dataset	***Percentile***	***2 variables (tide, NTR)***			***5 variables (tide, seiche, storm surge, PAW surge, IDAS)***		
		ESL Dep	***ESL Ind***	***NLI***	***ESL Dep***	***ESL Ind***	***NLI***
99th	99th	1.14	1.40	*-0.26*	1.13	1.36	*-0.23*
	99.5th	1.21	1.53	*-0.32*	1.18	1.43	*-0.25*
	99.9th	1.33	1.74	*-0.41*	1.28	1.61	*-0.33*
99.5th	99th	1.22	1.50	*-0.28*	1.22	1.51	*-0.29*
	99.5th	1.27	1.64	*-0.37*	1.27	1.59	*-0.32*
	99.9th	1.42	1.93	*-0.51*	1.45	1.77	*-0.32*
99.9th	99th	1.43	1.80	*-0.37*	1.40	1.76	*-0.36*
	99.5th	1.50	1.92	*-0.42*	1.47	1.83	*-0.36*
	99.9th	1.73	2.25	*-0.52*	1.56	2.01	*-0.45*

Supplementary Table S2. Return periods of the three most severe sea-level events according to the 99th, 99.5th, 99.9th datasets and computed using the univariate fit of ESLs with a Gamma distribution.

Event	***ESL dataset***	***RP (year)***
1966-11-04 (1.73 m)	99th	929
	99.5th	255
	99.9th	356
1979-12-22 (1.44 m)	99th	65
	99.5th	30
	99.9th	40
2019-11-12 (1.55 m)	99th	179
	99.5th	68
	99.9th	92

Extreme value theory: Hazard Scenarios and Copulas

This appendix provides general information on the theory supporting the extreme value analysis carried out in this study. A complete description is available in Salvadori et al.[1] and the other articles that are cited in the text.

Definition 1. *Let **X** be a random vector describing the phenomenon of interest. A Hazard Scenario of level* $\alpha \in (0, 1)$ *is any Upper Set* $S \subseteq \boldsymbol{R}^d$ *such that the relation*

$$P(\boldsymbol{X} \in S) = \alpha \qquad (1)$$

holds, where S is an Upper Set if, and only if, (i) $x \in S$ *and (ii)* $y \geq x$ *componentwise imply* $y \in S$.

Such a definition of Hazard Scenario is useful both for univariate and multivariate analyses. In particular, Return Periods (RP) are traditionally used to carry out frequency analyses. Interestingly enough, RP's (both univariate and multivariate ones) can well be associated with specific HS's[2,3].

Definition 2. *The Return Period T associated with the Hazard Scenario S is*

$$T = \mu / P(\boldsymbol{X} \in S), \qquad (2)$$

where μ *is the average inter-arrival time between successive occurrences* $\boldsymbol{X}_i$*'s of the phenomenon investigated.*

On the one hand, μ rules the time scale in which T is expressed (e.g., years). On the other hand, S is a subset of $\boldsymbol{R}^d$, and hence T simply "measures" the (average) frequency with which $\boldsymbol{X}$ takes value in S. Clearly, the denominator in Eq. (2) corresponds to the level α of S. In particular, in the univariate case, typically $S = \{x \in R: x > x^*\}$, where x^* is a suitable critical threshold of interest.

The multivariate analyses carried out in the present work are based on the mathematical Theory of Copulas[4,5]. A copula C is a d-dimensional function, with support on the hypercube $[0, 1]^d$ and image in $[0, 1]$, able to model the statistical dependence (or independence) between a set of d variables of interest, with $d = 2, 3, \ldots$ In particular, the Sklar Representation Theorem[6] states that any multivariate distribution function F can be written in terms of the copula C of the variables at play and the corresponding marginal laws F_i's (unicity is guaranteed if the marginals are continuous):

$$F_{X1,\ldots,Xd}(x_1, \ldots, x_d) = P(X_1 \leq x_1, \ldots, X_d \leq x_d) = C(u_1, \ldots, u_d), \qquad (3)$$

where $u_i = F_i(x_i) = P(X_i \leq x_i) \in [0, 1]$, with $i = 1, \ldots, d$.

The very first step in any multivariate analysis is to check the *degree of association* (viz., concordance or discordance) between pairs of variables. This is usually carried out by estimating the Kendall τ statistic and its statistical significance: traditionally, if the p-value of the corresponding independence test is smaller than 5%, then the two variables are regarded as dependent, otherwise, they are considered as independent. Kendall's rank correlation returns values between -1 and 1, where 0 typically indicates no dependence and 1 (-1) a perfect agreement (disagreement).

Should two variables be regarded as dependent, then a number of parametric copulas[4,5] could be used to model their dependence structures: free "R" software packages are available to fit, test, and select several families of copulas[7,8] corresponding to different kinds of dependence. In the present work, various investigations are carried out. The target is to compare the behaviour of specific aggregations (sums) of some observed variables determining ESL's with the aggregations of the same variables when the latter is modelled according to different dependence structures (copulas). Suitable Monte Carlo techniques are used to generate samples featuring the desired dependence properties.

Thanks to Sklar Representation Theorem[6], in order to construct a full statistical model of the random behaviour of the variables of interest, it is necessary and sufficient to individuate the marginal

distribution functions of the single variables at play, as well as their copula(s). For the univariate and copula fits, the following procedure is used:

1. Several standard univariate distributions (namely, Exponential, Lognormal, Loglogistic, Gamma, Chi-square, Fisher-Snedecor, Weibull, GEV, GPD) are fitted over the available data via a Maximum Likelihood procedure: these provide the marginal probability laws. In particular, only those distributions passing a Goodness-of-Fit test of Kolmogorov-Smirnov type (concerning the "body" of the distribution) or of Anderson-Darling type (concerning the "tails" of the distribution) are considered as admissible: here the GoF p-values are corrected for multiple testing. Then, among the admissible laws, a "best" one is chosen according to a (corrected) Akaike Information Criterion.
2. In case only pairs of variables are considered at a time, the following families of Copulas have been considered[4,5,7], including their rotated and flipped versions[7], amounting to about forty different dependence structures:
    - Archimedean (Ali-Mikhail-Haq, Clayton, Frank, Gumbel-Hougaard, Joe);
    - Elliptical (Student-t and Gaussian);
    - Extreme Value (Galambos, Husler-Reiss, Tawn, Gumbel);
    - Special (Farlie-Gumbel-Morgenstern, Plackett).

   The parameters are fitted via a Maximum Likelihood procedure. Then, admissible copulas are individuated via a Cramér-von Mises Goodness-of-Fit test (with p-values corrected for multiple testing), the most suitable one indicated for multivariate structures[9], and among the admissible ones, a "best" dependence structure is chosen according to a (corrected) Akaike Information Criterion.

3. In case that more than two variables are considered at a time, the corresponding multivariate dependence model is constructed by using Vine Copulas[10,8]. The admissibility of the multivariate copula is checked via a Cramér-von Mises Goodness-of-Fit test (adopting a Monte Carlo procedure[9]).

References

1. Salvadori, F. Durante, R., De Michele, C., Bernardi, M. and Petrella, L. A multivariate Copula-based framework for dealing with Hazard Scenarios and Failure Probabilities. *Water Resour. Res.*, 52(5):3701–3721 (2016). DOI: 10.1002/2015WR017225.
2. Salvadori, G. and De Michele, C.. Frequency analysis via copulas: theoretical aspects and applications to hydrological events. *Water Resour. Res.* 40:W12511 (2004). DOI: 10.1029/2004WR003133.
3. Salvadori, G., De Michele C., and Durante, F.. On the return period and design in a multivariate framework. *Hydrol. Earth Syst. Sci.* 15:3293-3305 (2011). DOI: 10.5194/hess-15-3293-2011.
4. Nelsen, R.B. An introduction to copulas. Springer-Verlag, New York, second edition (2006).
5. Salvadori, G., De Michele, C., Kottegoda, N.T. and Rosso, R.. Extremes in Nature. An approach using Copulas, volume 56 of Water Science and Technology Library Series. Springer, Dordrecht (2007). ISBN: 978-1-4020-4415-1.
6. Sklar, A. Fonctions de répartition à n dimensions et leurs marges. Publ. Inst. Statist. Univ. Paris, 8:229–231 (1959).
7. Hofert, M., Kojadinovic, I., Maechler, M. and Yan. J. Copula: Multivariate Dependence with Copulas. R package version 1.0-1 (2020).
8. Nagler T. and Vatter T. rvinecopulib: High Performance Algorithms for Vine Copula Modeling, R package version 0.5.5.1.1 (2021).

9. Genest, C., Rémillard, B., Beaudoin, D. Goodness-of-fit tests for copulas: A review and a power study. *Insurance: Mathematics and Economics*, 44:199–213 (2009).
10. Aas, K., Czado, C., Frigessi, A., Bakken, H. Pair-copula constructions of multiple dependence. *Insurance, Mathematics and Economics*, 44:182–198 (2009).
